# Strong, Biodegradable Lignocellulosic Films as Potential Bioplastics

**DOI:** 10.3390/polym18111359

**Published:** 2026-05-29

**Authors:** Zhenzhen Zhang, Ziyu Duan, Juan Wang, Jungang Jiang, Zhishun Wei, Silong Wu, Jan-Michael Albina

**Affiliations:** 1 Hubei Provincial Key Laboratory of Green Materials for Light Industry and New Materials and Green Manufacturing Talent Introduction and Innovation Demonstration Base, Hubei University of Technology, Wuhan 430068, China; 2110412427@hbut.edu.cn (Z.Z.); jungang.jiang@hbut.edu.cn (J.J.); wei.zhishun@hbut.edu.cn (Z.W.); 102210509@hbut.edu.cn (S.W.); 2 Shenzhen Institutes of Advanced Electronic Materials, Shenzhen Institutes of Advanced Technology, Chinese Academy of Sciences, Shenzhen 518055, China; zy.duan@siat.ac.cn

**Keywords:** lignocellulosic films, nanocellulose, mechanical properties, biodegradable, sustainability

## Abstract

Lignocellulosic films (LCFs) derived from biomass have attracted increasing attention owing to their abundant availability, recyclability, and biodegradability, making them promising candidates for replacing non-biodegradable plastics. Notably, the mechanical properties and wet stability of these materials play a crucial role in their practical applications. In this paper, we employ an eco-friendly and straightforward approach to synthesizing high-strength LCF by mixing nanocellulose with lignin. The incorporation of lignin enhances the mechanical strength for LCF, achieving a yield strength of 157.12 MPa at a lignin content of 15 wt% while simultaneously imparting excellent water absorption properties. Fourier transform infrared (FTIR) and contact angle measurements confirmed the structural integrity and hydrophilicity of the composite films. Excessive lignin content led to reduced mechanical performance, emphasizing the importance of optimizing the lignin-to-cellulose ratio. Therefore, this paper demonstrates the significant potential of LCF in developing environmentally friendly materials for applications in water treatment, packaging, flexible electronics, energy storage, and agriculture.

## 1. Introduction

With increasing environmental pollution and the depletion of fossil resources—where, without systemic interventions, an estimated 710million metric tons of plastic waste will cumulatively enter aquatic and terrestrial ecosystems by 2040 [1], imposing massive economic burdens with environmental damages projected to reach up to USD 281 trillion [2]—the development of biodegradable and environmentally benign materials as alternatives to conventional petroleum-based plastics is essential [3]. In particular, the serious soil pollution caused by conventional polyethylene mulching films underscores the urgent need for biodegradable substitutes, thereby creating significant opportunities for the application of lignin-based composites in agricultural mulching materials [4,5]. Nanocellulose fibers possess excellent mechanical strength, low density, and versatile modifiability along with a high specific surface area and outstanding reinforcing capabilities, making them highly promising for composite materials [6,7,8]. Lignin, as the second most abundant biopolymer in nature, is characterized by a complex and heterogeneous structure and abundant availability, and it can be effectively valorized via diverse depolymerization strategies [9]. As a naturally hydrophobic material with intrinsic adhesive properties, lignin may impart antioxidative, UV shielding, and antimicrobial functionalities to composites. Moreover, its integration with cellulose effectively enhances the hydrophilicity, thermal stability, and mechanical performance of pure cellulose-based materials [10,11]. Lu et al. comprehensively reviewed the preparation and applications of lignin micro- and nanoparticles across diverse fields, including energy, catalysis, agriculture, food, safety, and biomedicine [12]. Sulis et al. report the conversion of lignocellulosic feedstocks into bio-based energy and products as sustainable alternatives to fossil fuel-derived products [13].

Interestingly, Ewulonu et al. synthesized the lignocellulose nanofibers by adjusting the lignin content [14]. Zor et al. reported that the incorporation of 2.5 wt% nanolignin resulted in high thermal stability, enhanced mechanical strength, and a suitable morphological structure [15]. Nevarez et al. reported that the cellulose triacetate films incorporating 1 wt% lignin improves mechanical properties, yielding the highest Young’s modulus [16]. It was reported that incorporating lignin into cellulose matrices can fill voids within the fibrous network through interfacial interactions, such as hydrogen bonding and hydrophobic interactions, thereby densifying the material structure and significantly enhancing both the dry and wet mechanical strengths of the composite films [17]. Bio-based polymers, including polylactide, polyhydroxyalkanoates, polyhydroxybutyrate, poly (ε-caprolactone), polybutylene succinate, and natural fibers such as bamboo and banana fibers, have attracted increasing attention, providing a sustainable foundation for the development of LCFs [18,19].

However, there are some performance limitations, such as relatively low strength and low resistance [20]. Nanocellulose-based composites have broad applications in papermaking, packaging, automotive, and construction sectors; however, their large-scale utilization is limited by high production costs, complex processing requirements, high viscosity, and challenges in designing efficient dewatering and retention systems [21]. Furthermore, the degradation behavior of bioplastics is of significant importance [22]. Lignin-based nanomaterials have been widely applied in sustainable agriculture for the controlled release of fertilizers and pesticides, the modulation of plant responses [23], and as biodegradable alternatives to conventional plastic mulches [24]. The impact of recycling, reusing, and final disposal methods of composite films on the environment are the key factors for the biodegradable polymers. For example, research on biodegradable polymers has shown that selecting appropriate molecular structures can control their biodegradation rate and make them compatible with natural degradation mechanisms [25,26].

It is necessary to consider how the mechanical properties and durability of composite films are affected by environmental factors such as PH [27], temperature, humidity, microbial biomass [28], ultraviolet radiation [29], etc. These factors not only affect the service life of composite films but may also affect their environmental performance during use. Wu et al. synthesized cellulose nanofibrils containing 12 wt% lignin, exhibiting a tensile strength exceeding 31 MPa and a Young’s modulus greater than 3.9 GPa [30]. Abdel Aziz et al. synthesized carboxylated lignin-containing cellulose nanofibers via a nitro-oxidation process followed by high-pressure homogenization [31]. Dong et al. fabricated nanocellulose/lignin films through a layer-by-layer assembly approach, resulting in enhanced mechanical strength [32].

Further modifications utilizing lignin and cellulose have been reported. Biodegradable chitosan/PVA films were reinforced with nanocrystalline cellulose or nano-lignocellulose, demonstrating that optimizing filler–matrix combinations, rather than increasing nanocellulose content, enables simultaneous improvement in barrier and mechanical properties, offering a scalable route to transparent, high-performance, compostable food packaging materials [33]. It is reported that incorporating dual HCl and citric acid crosslinking, modified lignocellulosic nanofibers from bamboo shoot shells into polyvinyl alcohol films enhances mechanical properties, thermal stability, and barrier performance while reducing water sensitivity, owing to improved interfacial adhesion and uniform dispersion [34]. Cellulose dissolution systems enable the fabrication of regenerated cellulose (RC) films with high transparency, barrier performance, and biodegradability. Ryu et al. prepared three types of aqueous dissolution systems, including glycol ether/sodium hydroxide (NaOH), poly(ethylene glycol) (PEG)/NaOH, and urea/NaOH aqueous systems with lignocellulosic microfine solutions to synthesize the regenerated cellulose films. The dissolution yields ranged from 77.0% to 85.0%, while glycol-based co-solvents improved film transparency (>70%). Notably, PEG/NaOH systems exhibited superior solution stability, minimal viscosity change over 30 days, the lowest shrinkage (19.4%), and the highest mechanical strength (47.8 MPa), outperforming glycol ether/NaOH and urea/NaOH systems [35]. Alfalfa lignocellulosic extract is reported as an eco-friendly alternative to plastics. The optimized film showed moderate tensile strength, good flexibility, low water vapor permeability, strong UV–Vis–IR blocking ability, and antioxidant activity. It also demonstrated rapid biodegradability (over 90% within 29 days) [36]. Lignocellulose esters are promising renewable alternatives to petroleum-based plastics, but most studies focus on purified cellulose. Froment et al. performed a one-pot, room-temperature synthesis of cinnamate and benzoate esters from both microcrystalline cellulose and raw pine sawdust. Pine-derived esters show superior mechanical performance (5–8 MPa vs. 1–3 MPa), which is likely due to lignin-induced π–π interactions. The films are hydrophobic, thermoplastic, and suitable for packaging [37].

In this paper, we investigated the surface morphology and mechanical properties of LCFs with various mass fractions of lignin. Through rational strategies, these films have demonstrated exceptional performance and significant potential for applications as bioplastics.

## 2. Materials and Methods

### 2.1. Materials

The natural plant materials used in this paper were procured between June and August 2024 from a wood factory located in Hubei Province, China. Analytical-grade oxalic acid dihydrate and 1,4-dioxane were obtained from Sinopharm Chemical Reagent Co., Ltd. in Shanghai, China. Solutions were prepared with deionized water with the resistance of 18.2MΩ·cm at ${25\;}^{{}^{\circ}}C$ supplied by a Molecular Lab water ultra-purifier (Shanghai Molecular Scientific Instruments Co., Ltd., Shanghai, China).

### 2.2. Synthesis of Lignocellulosic Film (LCF)

**Figure 1:**
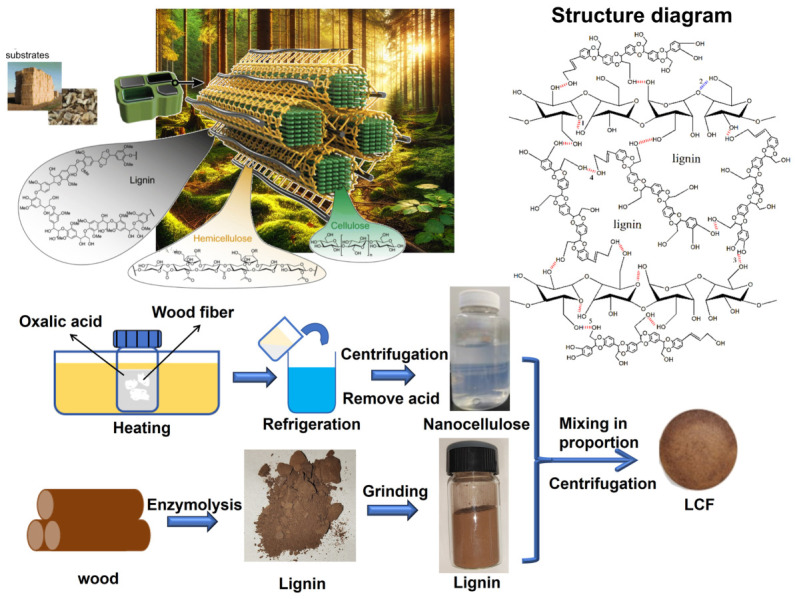
Schematic diagram of the preparation process for lignocellulosic films.

Figure 1 schematically illustrates the fabrication process of the lignocellulosic film (LCF), highlighting a fully bio-based and sustainable approach. All precursors used in this paper are renewable, plant-derived biopolymers, emphasizing the eco-friendly nature of the process. Lignin was first extracted from wood chips through enzymatic hydrolysis, which is a mild and selective method that preserves the polymer’s functional groups, and then further processed into a fine powder to ensure uniform dispersion within the composite matrix. Concurrently, nanocellulose was obtained via controlled oxalic acid hydrolysis at elevated temperatures, which efficiently breaks down cellulose fibers into nanoscale fibrils. After the hydrolysis, the reaction mixture was cooled, and residual acid and by-products were thoroughly removed through repeated filtration, yielding a stable, homogeneous nanocellulose dispersion suitable for film formation. This carefully controlled preparation of lignin and nanocellulose ensures high purity, optimal particle size, and excellent compatibility between the components, which are critical for achieving uniform morphology, enhanced mechanical performance, and superior barrier properties in the resulting LCF.

Lignin powder was obtained using a novel lignin isolation method based on double ball milling combined with enzymatic hydrolysis (DEL), as reported by Chen et al. [38], which is designed to maximize yield and preserve functional groups. Initially, the raw wood material was pre-extracted with benzyl alcohol to remove low-molecular-weight extractives and other soluble impurities, ensuring a cleaner starting substrate. The pretreated material then underwent planetary ball milling for 60 h, which is a mechanical process that significantly reduces particle size and disrupts the lignocellulosic structure, enhancing accessibility for subsequent enzymatic hydrolysis. The resulting fine powder was dispersed in a sodium acetate buffer (pH = 4.8) at a solid-to-liquid ratio of 1:20 (g·mL^−1^), after which a cellulose enzyme (50 FPU g^−1^ substrate) was added to initiate hydrolysis. Enzymatic treatment was conducted in a water-bath shaker at 50 °C for 48 h, allowing for the selective degradation of cellulose while leaving lignin largely intact. Following hydrolysis, the suspension was centrifuged to separate the solid residue from soluble sugars and degraded cellulose. The solid fraction was thoroughly washed with sodium acetate buffer (pH = 4.8) to remove residual enzymes and soluble hydrolysis products, after which it was freeze-dried to stabilize the material. To further enhance lignin purity and reduce particle size, the dried residue underwent a second ball-milling treatment for 24 h, which was followed by repeated enzymatic hydrolysis under identical conditions to remove remaining polysaccharides. The final solid residue was carefully washed with acidified water (pH = 2.0) to eliminate any remaining salts and weakly bound sugars; then, it was freeze-dried and finely ground to yield a uniform lignin powder, which is suitable for incorporation into lignocellulosic films. This DEL procedure ensures high lignin purity, fine particle size, and retention of functional groups, which are critical for effective dispersion and interaction with other biopolymers in composite films.

Oxalic acid was first heated to its molten state at 110 °C to ensure complete melting and uniform heat distribution. Bleached coniferous wood pulp was then gradually added to the molten acid at a solid-to-liquid ratio of 1:50, allowing the fibers to be fully exposed to the reactive medium. The mixture was maintained under constant stirring at 110 °C for 30 min, facilitating controlled hydrolysis of the cellulose fibers into nanofibrils while partially depolymerizing amorphous regions. Upon completion of the reaction, 100–150 mL of warm water was promptly added to quench the reaction, preventing over-hydrolysis and thermal degradation of the cellulose. The resulting mixture was filtered through a 0.45 μm membrane to separate the solid nanocellulose (NC) from the acidic supernatant, after which it was washed 3–5 times with deionized water to remove residual oxalic acid and soluble by-products. The collected precipitate was redispersed in water to a concentration of approximately 0.5 wt%, forming a stable suspension. To further purify the NC, the suspension was transferred into a dialysis bag and immersed in deionized water for 7 days with daily water replacement to remove residual acid and low-molecular-weight impurities, ensuring chemical neutrality and high colloidal stability. Finally, the purified suspension was subjected to ultrasonic homogenization, which broke down any remaining aggregates and produced a uniform NC dispersion with the desired nanoscale dimensions, which is suitable for subsequent incorporation into composite films or other functional materials.

The NC dispersion was then carefully combined with the prepared lignin powder in a clean beaker with the total solid content (NC + lignin) maintained at 60 mg to ensure consistency across all samples. Lignin was incorporated at varying loadings of 0, 5, 10, 15, 20, and 25 wt% to systematically investigate its effect on the properties of the resulting LCF. To facilitate uniform lignin distribution within the NC matrix, an appropriate volume of deionized water and a small amount of 1,4-dioxane were added, serving as co-solvents to enhance lignin dispersibility. The mixture was then subjected to ultrasonic treatment for sufficient time until the lignin was thoroughly dispersed, and no visible aggregates were observed, ensuring a homogeneous suspension at the nanoscale. The well-dispersed suspension was subsequently processed using vacuum-assisted filtration. As the liquid phase was drawn through the microporous membrane, the NC and lignin components gradually accumulated on the membrane surface, forming a coherent, self-supporting LCF. After the filtration was complete and the excess liquid was removed, the wet film was allowed to dry naturally at ambient conditions, which minimized internal stresses and preserved structural integrity. The partially dried film was then gently peeled from the membrane to avoid mechanical damage and further dried in a vacuum oven to remove residual moisture, yielding the final LCF with uniform thickness, smooth surface, optical transparency, and consistent mechanical and barrier properties. This carefully controlled procedure ensured high-quality composite films suitable for subsequent characterization and application studies.

### 2.3. Structural Characterization

The particle size, distribution, and surface morphologies of the samples were characterized by field emission scanning electron microscopy (FE-SEM, SU8010, Hitachi, Japan) at an acceleration voltage of 3kV. Prior to observation, the sample surfaces were sputter-coated with a thin layer of Au to improve electrical conductivity. Fourier transform infrared (FTIR) spectra were recorded using an INVENIO-S Tensor II spectrometer (Bruker Optics Pty. Ltd., Billerica, MA, USA). Nanocellulose dispersion, lignin powder, LCF-5, LCF-10, LCF-15, LCF-20 and LCF-25 films were analyzed over the wavenumber range of 400–4000 cm^−1^ using a diamond ATR crystal. Measurements spectra were collected under a protective N^2^ atmosphere with 16 scans at a resolution of 4 cm^−1^. X-ray diffraction (XRD) patterns were collected on an X-ray diffractometer (Empyrean, PANalytical B.V., Almelo, The Netherlands) to characterize the crystal structure and phase composition of lignin powder, LCF-5, LCF-10, LCF-15, LCF-20 and LCF-25. The instrument operated using a Cu Kα source (λ=0.15418 nm) at 40 kV and 40 mA. Data acquisition was performed from 5° to 90° (2θ) with a step size of 0.013° and a counting time of 10 s per step. HighScore Plus software (version 5.3.1, PANalytical B.V., The Netherlands) was utilized for subsequent data processing and analysis. The surface elemental composition and chemical states of the samples were analyzed by X-ray photoelectron spectroscopy (XPS, PHI 5000 VersaProbe III, ULVAC-PHI Inc., Chigasaki, Japan). The high-resolution XPS spectra were processed, deconvoluted, and peak-fitted using CasaXPS software (version 2.3.26 PR1.0, Casa Software Ltd., Teignmouth, UK).

### 2.4. Mechanical Properties

Static water contact angles were measured using a JC2000D Contact Angle Measuring Instrument (Shanghai Zhongchen Digital Technology Equipment Co., Ltd., Shanghai, China). A 1 μL droplet of deionized water was deposited on the surface of each film, and six measurements were taken per sample to account for surface heterogeneity.

Tensile properties were evaluated using a CMT4204 Electronic Tensile Testing Machine (MTS Systems Co., Ltd., Shanghai, China) following the ISO 1184-1983 standard for plastic films and sheets thinner than 1 mm. The entry force was set to 0.01 N, and a 0.8 kN load cell was used to record the force required for sample failure. The crosshead speed was maintained at 10.0 mm·min^−1^ with force and displacement continuously recorded via a PC. The magnitude of constant force decay was below 40%.

The tensile stress (σ) and strain (ϵ) were calculated according to Equations (1) and (2):

(1) $$\sigma =\frac{F}{A}$$

where σ is the normal stress (MPa), *F* is the normal force (N) acting perpendicular to the area, and *A* is the area (mm).

(2) $$\epsilon =\frac{\Delta L}{L_{0}}\times 100\%$$

where ϵ is normal strain, ΔL is the change of length (mm), and $L_{0}$ is the initial length (mm).

## 3. Results and Discussion

### 3.1. Surface Properties and Structural Analysis

**Figure 2:**
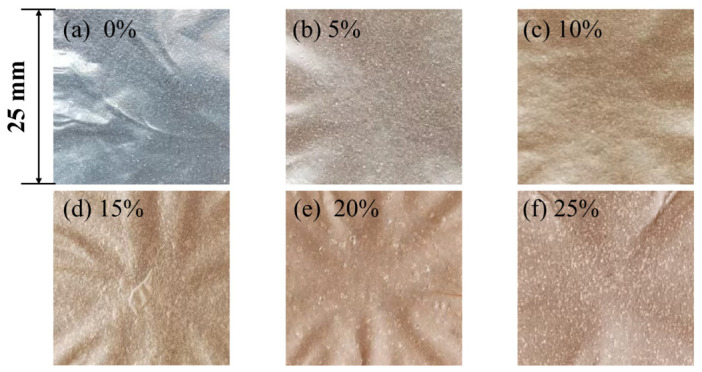
Lignocellulosic films with various lignin mass fractions: (**a**) nanocellulose (NC), (**b**) LCF-5 (lignin 5 wt%), (**c**) LCF-10 (lignin 10 wt%), (**d**) LCF-15 (lignin 15 wt%), (**e**) LCF-20 (lignin 20 wt%), (**f**) LCF-25 (lignin 25 wt%).

Figure 2 shows the LCF samples with varying lignin contents: (a) nanocellulose (NC), (b) LCF-5 (5 wt% lignin), (c) LCF-10 (10 wt% lignin), (d) LCF-15 (15 wt% lignin), (e) LCF-20 (20 wt% lignin), and (f) LCF-25 (25 wt% lignin) after vacuum filtration. As the lignin content increases, the films gradually change in appearance from transparent silver to increasingly brown and less transparent, reflecting the effective incorporation of lignin into the lignocellulosic matrix. This color shift is not only a visual indicator of lignin presence but also suggests alterations in the film’s microstructure and light-scattering behavior, which is likely due to lignin’s aromatic structure and tendency to form aggregates. The progressive darkening correlates with increased lignin content, which can influence optical properties, reduce transparency, and potentially affect other functional characteristics such as UV absorption and barrier performance.

**Figure 3:**
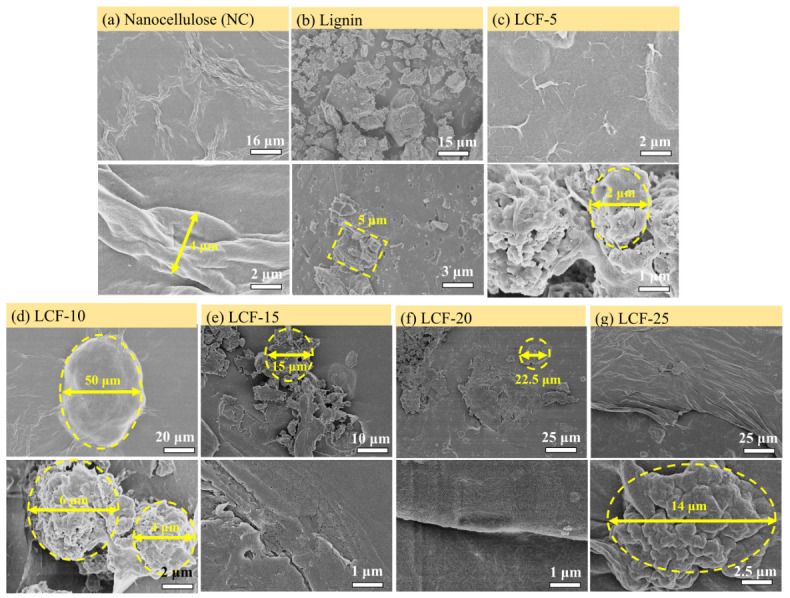
FE-SEM micrographs of the samples: (**a**) nanocellulose (NC) liquid; (**b**) pure lignin powder. FE-SEM micrographs of the composite films: (**c**) LCF-5, (**d**) LCF-10; (**e**) LCF-15; (**f**) LCF-20; (**g**) LCF-25.

Figure 3 presents the surface morphologies of the samples characterized by field-emission scanning electron microscopy (FE-SEM): (a) nanocellulose (NC) suspension, (b) lignin powder, (c) LCF-5, (d) LCF-10, (e) LCF-15, (f) LCF-20 and (g) LCF-25, respectively. As shown in Figure 3a, the NC exhibits well-defined nanocrystal-like structures with lengths ranging from 1 to 4 μm, forming a relatively interconnected and entangled network. This network provides a strong and flexible framework that is crucial for supporting additional components, such as lignin, within the composite matrix. In Figure 3b, several dark, roughly square-shaped particles can be observed, which are presumed to be lignin aggregates with diameters of approximately 1∼5 μm. These particles indicate that prior to incorporation into the composite, lignin can exist as micro-scale aggregates. Figure 3c presents the morphology of the LCF-5 sample, in which dark, irregularly shaped particles with diameters ranging from 1 to 2 μm are embedded within the nanocellulose network. The relatively small size and even distribution of these particles suggest that at low lignin loading (5 wt%), lignin can be incorporated into the cellulose matrix with minimal aggregation, forming a relatively homogeneous composite structure. This uniform dispersion is expected to contribute positively to mechanical integrity and barrier properties. As the lignin content increases, the surface morphology of the films changes markedly, as illustrated in Figure 3d–g. In the LCF-10 film (Figure 3d), the surface remains relatively smooth and uniform, indicating that lignin is still well dispersed within the NC matrix at this moderate loading. However, further increases in lignin content to 15 wt% (Figure 3e) and 25 wt% (Figure 3g) result in a progressively rougher surface. At these higher loadings, small agglomerates with diameters of approximately 15 μm and 22.5 μm begin to appear, indicating a partial aggregation of lignin particles. The self-aggregation behavior of lignin observed at elevated concentrations (>15wt%) is intrinsically governed by its specific extraction procedure and the resulting molecular structuration. In contrast to traditional harsh industrial isolation techniques, the double ball milling combined with enzymatic hydrolysis (DEL) method utilized in this paper represents a highly mild physical–biological approach. As demonstrated in recent structural analyses of similar mild extraction protocols (e.g., milled wood lignin) [39], these methodologies effectively preserve the native macro-molecular architecture of lignin, maintaining a high degree of polymerization, intact β-O-4 linkages, and a high density of functional aromatic rings. While this structural integrity facilitates strong hydrogen-bonding interactions with the nanocellulose matrix at optimal loadings (≤15wt%), it concurrently preserves the strong inherent cohesive forces of the lignin macromolecules. According to recent literature discussing lignin structuration [40], the abundant and structurally intact aromatic moieties in such native-like lignin intrinsically promote strong non-covalent interactions. Consequently, when the lignin concentration exceeds the critical threshold of 15wt%, this intermolecular self-affinity thermodynamically surpasses the interfacial adhesion (lignin–cellulose hydrogen bonding). This structural competition drives the highly preserved lignin macromolecules to self-assemble, inevitably leading to the formation of the micro-scale agglomerates observed in the FE-SEM images of LCF-20 and LCF-25. The formation of these aggregates can influence the optical, mechanical, and barrier properties for LCF, as regions with higher lignin content may act as stress concentration points or affect the uniformity of gas and moisture diffusion through the film. These observations are highly consistent with recent findings in similar biocomposite systems. For instance, studies by Zor et al. [15] and Bai et al. [17] similarly demonstrated that while moderate lignin incorporation enhances the network’s density and water stability, excessive bio-filler content inevitably disrupts the continuity of the nanocellulose framework. Furthermore, the strong self-aggregation tendency of lignin at high concentrations creates interfacial micro-voids, which act as preferential pathways that accelerate moisture diffusion. Hence, the SEM observations demonstrate that lignin content must be carefully controlled to balance uniform dispersion and maintain the structural integrity of the LCF.

**Figure 4:**
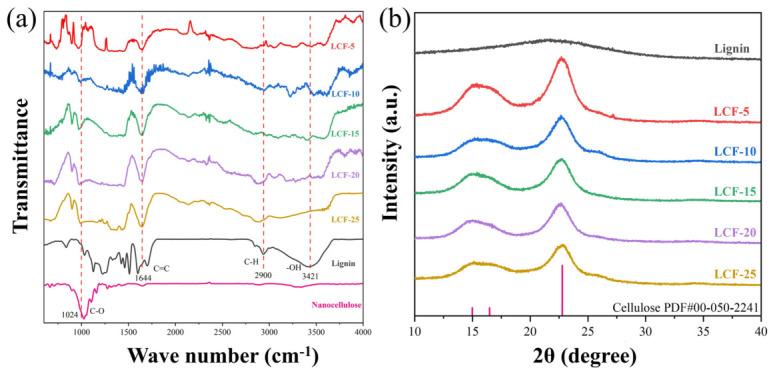
(**a**) FTIR spectroscopy of nanocellulose suspension, lignin powder, LCF-5, LCF-10, LCF-15, LCF-20 and LCF-25; (**b**) XRD patterns of lignin powder, LCF-5, LCF-10, LCF-15, LCF-20 and LCF-25.

Fourier transform infrared (FTIR) spectra of NC, lignin, LCF-5, LCF-10, LCF-15, LCF-20 and LCF-25 are presented in Figure 4a, providing insight into the chemical structure and interactions within the composite. All LCF composite films exhibited characteristic absorption bands corresponding to both cellulose and lignin, confirming the successful incorporation of lignin into the nanocellulose matrix. In the spectrum of pure NC, a characteristic peak appears around 1024.44 cm^−1^, corresponding to the C-O stretching vibration, which is the polysaccharide backbone from NC. For lignin powder, several characteristic peaks are observed in the 1125.56∼2000 cm^−1^ range, which are associated with aromatic ring vibrations and C-O-C linkages within the lignin structure. Additionally, a broad peak centered at 3421.07 cm^−1^ corresponds to the stretching vibration of hydroxyl groups, reflecting the abundance of -OH functionalities inherent to lignin. Compared with pure NC, all LCF composite films showed broadened and overlapped hydroxyl absorption bands in the region of 3330–3420 cm^−1^, indicating enhanced intermolecular hydrogen-bonding interactions between lignin and cellulose components. Moreover, the intensity of lignin-related absorption features gradually increased with increasing lignin content, suggesting the progressive incorporation of lignin into the cellulose matrix. No new characteristic absorption peaks were observed in the LCF spectra, indicating that no additional covalent chemical bonds were generated during composite film fabrication. Therefore, the interaction between lignin and nanocellulose is mainly attributed to physical entanglement and hydrogen-bonding interactions. These intermolecular interactions contribute to the improved interfacial compatibility and structural integrity within the composite films.

X-ray diffraction (XRD) measurements were conducted to investigate the crystalline structure of lignin/cellulose nanofiber (LCF) composite films with different lignin contents. As shown in Figure 4b, pure lignin exhibited a broad diffraction halo without distinct sharp peaks, indicating its predominantly amorphous nature. In contrast, all LCF composite films displayed characteristic diffraction peaks at approximately 2θ=15–$17^{\circ }$ and 22–$23^{\circ }$, which are assigned to the typical cellulose I crystalline structure (PDF#00-050-2241). The diffraction peak centered around $22.5^{\circ }$ corresponds to the (200) crystallographic plane of cellulose I, while the weaker peaks near 15–$17^{\circ }$ are associated with the $\left(1\bar{1}0\right)$ and (110) planes. These characteristic peaks demonstrate that the crystalline structure of nanocellulose was retained after the incorporation of lignin. With increasing lignin content from 5 wt% to 25 wt%, the intensity of the cellulose diffraction peaks gradually decreased and the diffraction peaks became broader, suggesting that the incorporation of amorphous lignin partially disrupted the ordered arrangement of cellulose molecular chains and reduced the overall crystallinity of the composite films. Nevertheless, no new diffraction peaks were observed, indicating that lignin incorporation did not alter the native cellulose I crystal structure. XRD analysis was therefore performed to evaluate the structural characteristics and crystallinity behavior of the nanocellulose within the lignocellulosic composite films.The crystallinity index values of the LCF composite films were calculated using the Segal method based on the XRD diffraction intensities. The CrI values of LCF-5, LCF-10, LCF-15, LCF-20, and LCF-25 were determined to be 70.01%, 64.99%, 68.30%, 72.94%, and 61.79%, respectively. The CrI values showed slight fluctuations with increasing lignin content, which may be attributed to the heterogeneous distribution of lignin and the rearrangement of cellulose molecular chains during film formation. Nevertheless, all composite films maintained relatively high crystallinity, indicating that the cellulose I crystalline structure was largely preserved after lignin incorporation.

**Figure 5:**
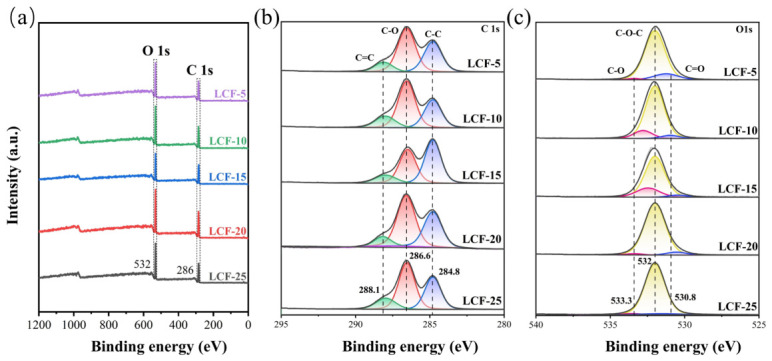
(**a**) Full of high-resolution X-ray photoelectron spectrum (XPS) of LCF; (**b**) high-resolution C 1*s* spectrum of LCF; (**c**) high-resolution O 1*s* spectrum of LCF.

Figure 5a presents the high-resolution X-ray photoelectron spectroscopy (XPS) survey spectrum of the LCF samples, offering detailed insight into the surface elemental composition and chemical states of the composite films. The spectrum reveals dominant peaks corresponding to C 1*s* at approximately 286.0 eV and O 1*s* at around 532.0 eV, confirming that carbon and oxygen are the primary elements in the LCF structure. The absence of additional peaks related to other elements indicates that the films are of high purity, with minimal contamination or residual processing agents, which is essential for ensuring reproducibility and consistent performance in potential applications such as packaging or adsorption. High-resolution spectra of C 1*s* and O 1*s* are presented in Figure 5b,c, providing further details on the chemical bonding environment. The C 1*s* spectrum can be deconvoluted into three distinct peaks at 288.1 eV, 286.6 eV, and 284.7 eV, corresponding to C=C, C-O, and C-C bonds, respectively. The presence of oxygen-containing functionalities, such as C-O bonds, indicates partial oxidation of the surface and the retention of hydroxyl groups from cellulose and lignin. These surface functionalities enhance intermolecular hydrogen bonding and improve interfacial adhesion between the lignin and NC components, which can directly influence the mechanical strength and barrier properties of the composite. Additionally, such oxygenated groups are advantageous for adsorption applications, as they provide active sites for interactions with polar molecules. The O 1*s* spectrum further confirms the chemical diversity within the LCF matrix, showing three characteristic peaks at 533.0 eV, 532.0 eV, and 530.8 eV, which are assigned to C-O, C-O-C, and C=O species, respectively. The relatively strong intensity of the C-O and C-O-C peaks indicates that the cellulose-derived polysaccharide structure is largely preserved in the films, maintaining the integrity of the NC network. The presence of C=O species may arise from lignin-derived aromatic oxidation or partial carbonization during processing, suggesting that lignin contributes additional functional groups that enrich the chemical complexity of the composite. Collectively, these XPS results confirm the coexistence of multiple oxygen-containing functionalities, demonstrating that the LCF exhibits a chemically heterogeneous surface capable of supporting strong intermolecular interactions, enhanced structural stability, and potential functional properties such as adsorption, barrier performance, or catalytic activity.

### 3.2. Mechanical Properties of LCF

**Figure 6:**
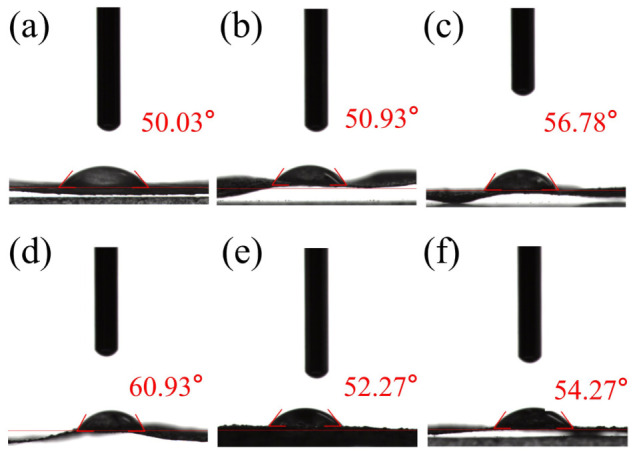
Contact angle information of the LCF with different lignin mass fractions: (**a**) NC, (**b**) LCF-5, (**c**) LCF-10, (**d**) LCF-15, (**e**) LCF-20, (**f**) LCF-25.

Figure 6 presents the water contact angle measurements of the LCF with varying lignin mass fractions: (a) 0 wt%, (b) 5 wt%, (c) 10 wt%, (d) 15 wt%, (e) 20 wt%, and (f) 25 wt%. As the lignin content increases, the contact angle of the composite films shows a moderate variation rather than a monotonic trend. Notably, the sample with a lignin mass fraction of 15 wt% exhibits the highest contact angle, reaching 60.93°, indicating a relatively reduced surface hydrophilicity at this composition. Although the contact angle reached a maximum of 60.93°, it remained below 90°, suggesting that the composite films maintain an overall hydrophilic character regardless of lignin content. This behavior can be attributed to the abundant hydroxyl groups derived from the nanocellulose matrix, which dominate the surface wettability even in the presence of hydrophobic lignin components. The enhanced hydrophilicity of the LCF contributes significantly to their improved mechanical properties. The abundant hydroxyl (-OH) groups on nanocellulose promote strong hydrogen bonding within the matrix and at the lignin–cellulose interface, leading to better interfacial adhesion and more efficient stress transfer. In addition, the hydrophilic nature helps achieve a more uniform dispersion of lignin, reducing structural defects and stress concentration points. The resulting dense and homogeneous structure enhances both strength and mechanical stability. Thus, the hydrophilicity not only affects surface wettability but also plays an important role in reinforcing the mechanical performance of the composite films.

**Figure 7:**
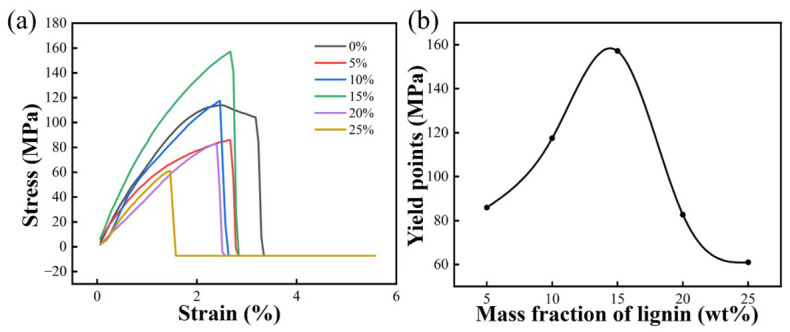
(**a**) Stress as a function of strain for LCF samples; (**b**) the yield point as a function of the mass fraction of lignin.

Tensile testing is a fundamental method for evaluating the mechanical properties of materials. Figure 7a shows the stress–strain curves of the LCF samples with varying lignin mass fractions. The tensile strength of the LCF samples ranges from 60 to 160 MPa, demonstrating that the LCF samples possess excellent mechanical properties. The lignin structure contains abundant functional groups that can serve as hydrogen bonding sites, thereby enhancing intermolecular interactions within the composite. In this paper, the incorporation of lignin aims to improve mechanical performance by strengthening hydrogen bonding interactions.

However, owing to the relatively large particle size and heterogeneous nature of lignin powder, excessive addition may disturb the nanocellulose network and weaken the van der Waals interactions that contribute to structural integrity. Therefore, it is essential to optimize the lignin content to achieve a balance between hydrogen bonding and van der Waals forces, thereby maximizing the overall mechanical performance of the composite films. Figure 7b shows the variation of yield strength with lignin mass fraction in the LCF samples. The yield strength increases with lignin content, reaching a maximum of 157.12 MPa at 15 wt%. Compared with other bioplastics in recent studies, the LCF-15 exhibits significantly superior mechanical performance. For instance, Wu et al. synthesized nanocellulose containing 12 wt% lignin with a tensile strength exceeding 31 MPa [30], and Ryu et al. reported regenerated cellulose films with a mechanical strength of 47.8 MPa [35]. Our unique double ball milling combined with enzymatic hydrolysis (DEL) method likely preserves critical functional groups, allowing for the exceptional 157.12 MPa yield strength observed in this paper. However, when the lignin content exceeds 15 wt%, the yield strength decreases. This decline may be attributed to excessive lignin incorporation, which can disrupt the continuity of the nanocellulose framework and weaken the structural cohesion of the composite. High lignin content may also cause non-uniform dispersion and reduce stress transfer efficiency, thereby compromising mechanical performance. Therefore, LCF-15 shows the most favorable mechanical properties among the tested samples. Interestingly, this maximum mechanical performance coincides with the peak contact angle of $60.93^{\circ }$ discussed in the previous section. While surface wettability and bulk mechanical strength are governed by distinct physical phenomena, their synchronized peaks at 15wt% lignin loading jointly reflect the optimal microstructural state of the composite. At this critical concentration, the highly uniform dispersion of lignin effectively reinforces the nanocellulose network within the bulk matrix, thereby enhancing structural strength, while concurrently ensuring an even distribution of relatively hydrophobic aromatic structures on the film surface to increase the contact angle. Consequently, when the lignin concentration exceeds 15wt%, the onset of agglomeration simultaneously creates stress concentration points that deteriorate mechanical integrity and disrupts the uniform surface chemistry, leading to the observed decline in both yield strength and contact angle.

## 4. Conclusions

LCF samples were successfully fabricated through the controlled incorporation of lignin into an NC matrix and subsequently subjected to systematic characterization to evaluate their structural, chemical, and functional properties. SEM analysis revealed that the films possess a compact, dense, and homogeneous microstructure, indicating that lignin particles are well dispersed and exhibit good compatibility with the NC network. This uniform distribution is critical for achieving consistent mechanical performance and minimizing stress concentration points that could lead to premature failure. FTIR and XPS analyses further confirmed the presence of abundant oxygen-containing functional groups, such as hydroxyl and ether groups within the films. These functional groups facilitate strong intermolecular interactions, particularly hydrogen bonding, between lignin and NC, which enhances cohesion within the film matrix and contributes to overall structural stability. The synergistic effect of these interactions is evident in the mechanical and surface properties of the composites. Notably, LCF-15 exhibited the highest yield strength of 157.12 MPa and a water contact angle of 60.93°, indicating a favorable balance between mechanical robustness and moderate hydrophilicity. These results suggest that an optimal lignin content reinforces the NC framework, improving structural integrity without compromising film flexibility. In contrast, excessive lignin loading leads to aggregation, uneven dispersion, and potential microstructural defects, which can diminish mechanical performance and reduce barrier or surface properties. LCF shows good mechanical strength and stability, making it promising for applications in packaging, agriculture, and the pharmaceutical field.

Despite the promising reinforcement outcomes achieved at optimal loadings, addressing the limitation of lignin agglomeration at higher concentrations (>15wt%) remains a critical challenge for maximizing the utilization of biomass. To overcome this limitation and further advance the field, our next strategic action lines will focus on the targeted surface modification of lignin (e.g., partial acetylation or esterification) to mitigate its inherent self-affinity, alongside the exploration of bio-based coupling agents to enhance interfacial compatibilization. Furthermore, comprehensive real-world degradation trials and pilot-scale extrusions will be prioritized to fully realize the commercial viability of these biocomposites in sustainable packaging and agricultural mulching applications. 

## Data Availability

Dataset available on request from the authors.

## Abbreviations

The following abbreviations are used in this manuscript:

LCF	Lignocellulosic films
NC	Nanocellulose
DEL	Enzymatic hydrolysis
FTIR	Fourier transform infrared
FE-SEM	Field emission scanning electron microscopy
RED	X-ray diffraction
